# Avian Influenza Outbreaks in Chickens, Bangladesh

**DOI:** 10.3201/eid1412.071567

**Published:** 2008-12

**Authors:** Paritosh K. Biswas, Jens P. Christensen, Syed S.U. Ahmed, Himel Barua, Ashutosh Das, Mohammed H. Rahman, Mohammad Giasuddin, Abu S.M.A. Hannan, Mohammad A. Habib, Abdul Ahad, Abu S.M.S. Rahman, Rayhan Faruque, Nitish C. Debnath

**Affiliations:** Chittagong Veterinary and Animal Sciences University, Chittagong, Bangladesh (P.K. Biswas, S.S.U. Ahmed, H. Barua, A. Das, M.H. Rahman, A. Ahad, R. Faruque, N.C. Debnath); Copenhagen University, Copenhagen, Denmark (J.P. Christensen); Bangladesh Livestock Research Institute, Dhaka, Bangladesh (M. Giasuddin); Department of Livestock Services, Dhaka (A.S.M.A. Hannan, M.A. Habib); Department of Livestock Services, Joypurhat, Bangladesh (A.S.M.S. Rahman)

**Keywords:** Epidemiology, avian influenza virus, H5N1, H9N2, chickens, Bangladesh, dispatch

## Abstract

To determine the epidemiology of outbreaks of avian influenza A virus (subtypes H5N1, H9N2) in chickens in Bangladesh, we conducted surveys and examined virus isolates. The outbreak began in backyard chickens. Probable sources of infection included egg trays and vehicles from local live bird markets and larger live bird markets.

The threat that highly pathogenic avian influenza (HPAI) A virus subtype H5N1 poses to poultry and public health has intensified (*1*). As the virus becomes established in poultry in developing countries, the number of human cases increases (*1*,*2*). Countries in the Asian Association for Regional Co-operation are especially vulnerable to virus perpetuation because of insufficient biosecurity, rearing of chickens and ducks together, selling of live birds, and deficient disease surveillance. To prevent human infection with avian influenza (H5N1), knowledge of avian influenza epidemiology is needed. We therefore describe the epidemiology of HPAI outbreaks in chickens in Bangladesh.

## The Study

Through July 10, 2007, we investigated 52 outbreaks caused by HPAI virus (H5N1) and 3 outbreaks caused by low-pathogenicity avian influenza (LPAI) virus (H9N2) in chickens in Bangladesh. After a high number of chicken deaths on a farm was reported to an *upazila* (a lower administrative unit of Bangladesh) veterinarian, the sick chickens on the farm were examined. From each of 55 outbreaks, 2 dead chickens were sent to a field disease investigation laboratory or to the Central Disease Investigation Laboratory, where oropharyngeal swabs were tested for avian influenza A virus antigen. From cases with positive results, tracheal samples were referred to the National Reference Laboratory for Avian Influenza (NRL-AI) for viral RNA extraction and purification (*3*), reverse transcription–PCR that used a primer set of hemagglutinin (H) genes (*4*), and end-product visualization. When NRL-AI confirmed H5, the farm was considered HPAI affected and was reported to the Department of Livestock Services. Tracheal samples from chickens involved in 37 outbreaks, including those that were A-antigen positive but H5 negative, were sent to the Veterinary Laboratory Agency in the United Kingdom for confirmation. A farm on which influenza subtype H9N2 was found was considered LPAI affected. All farms affected with HPAI or LPAI virus were called avian influenza–affected farms. A district or *upazila* with at least 1 avian influenza–affected farm was considered an infected district or infected *upazila.*

To collect information about the farms, we used a pretested questionnaire administered by 2 veterinarians. The form had space where veterinarians could add additional comments on the probable virus sources for infections by backward tracing (window <21 days of onset of clinical signs) and sources of spread by forward tracing (window between onset of clinical signs and culling), which they obtained by interviewing the affected farmers and allied personnel. Farm geographic coordinates were recorded. Through another questionnaire, we collected data on commercial and backyard farms and outbreaks from the *upazila* livestock offices. All avian influenza data stored at the Department of Livestock Services head office, field disease investigation laboratories, the Central Disease Investigation Laboratory, and NRL-AI were also collected.

Summary statistics were computed and plotted by using Excel (Microsoft, Redmond, WA, USA), Arc View 9.1 (Environmental Systems Research Institute, Redlands, CA, USA), and STATA 7 (Stata Corp., College Station, TX, USA). A 7-day rolling mean of avian influenza–affected farms, according to date of clinical onset of disease, was calculated from January 12, 2007, and plotted as a bar chart on day 4 for each value. Attack rates of farms were calculated separately for *upazilas* of every infected district (Table 1).

**Table 1:** Attack rates (infected *upazila*[*s*] for the infected districts) of avian influenza outbreaks in Bangladesh, 17/64 districts, January–July 2007

District	No. farms, infected/total*			Attack rate (95% CI)†	
	Commercial	Backyard		Commercial	Backyard
Dhaka	14/744	0/0		0.019 (0.027–0.009)	
Dinajpur	0/29	1/124,872			0.000008 (0.000018–0.000002)
Gaibandha	1/69	0/52,950		0.014 (0.041–0.009)	NA
Gazipur	3/1,956	0/0		0.001 (0.0032–0.0002)	NA
Jamalpur	5/144	0/58,962		0.034 (0.063–0.005)	NA
Jessore	2/298	3/331,295		0.006 (0.014–0.002)	0.000003 (0.000008–0.000002)
Joypurhat	0/124	1/101,152		NA	0.000009 (0.000027–0.00009)
Lalmonirhat	0/85	2/76,582		NA	0.00002 (0.00005–0.00001)
Magura	0/82	1/83,607		NA	0.00001 (0.00003–0.00001)
Naogaon	0/109	2/80,752		NA	0.00002 (0.00005–0.00001)
Narayangonj	7/925	0/0		0.007 (0.012–0.002)	NA
Nilphamari	0/119	5/277,984		NA	0.00001 (0.00003–0.00001)
Noakhali	1/97	0/0		0.010 (0.029–0.009)	NA
Rajbari	1/395	2/311,279		0.002 (0.006–0.002)	0.00006 (0.000014–0.000002)
Rangpur	0/95	2/143,263		NA	0.00001 (0.00003–0.00001)
Tangail	1/227	0/91,650		0.004 (0.012–0.004)	NA
Thakurgaon	0/1,137	1/73,391		–	0.00001 (0.00003–0.00001)
Total	35/5,635	20/1,807,739		0.006 (0.008–0.004)	0.00001 (0.000014–0.000006)

*Infected, avian influenza–infected farms; total, farms in the infected *upazila*(*s*) of a district. †CI, confidence interval; NA, not applicable.

**Figure 1:**
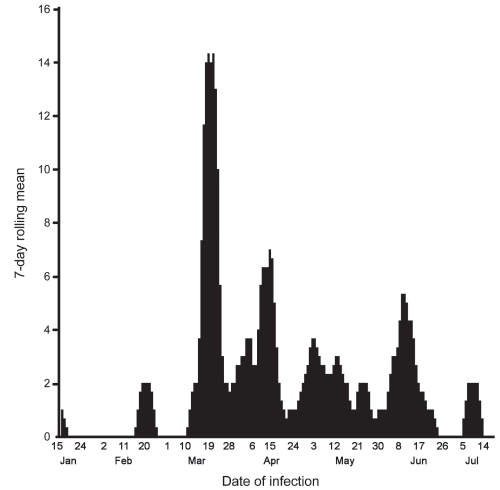
Seven-day rolling mean of occurrence of avian influenza–affected farms in outbreaks of highly pathogenic avian influenza, Bangladesh, January–July, 2007.

**Figure 2:**
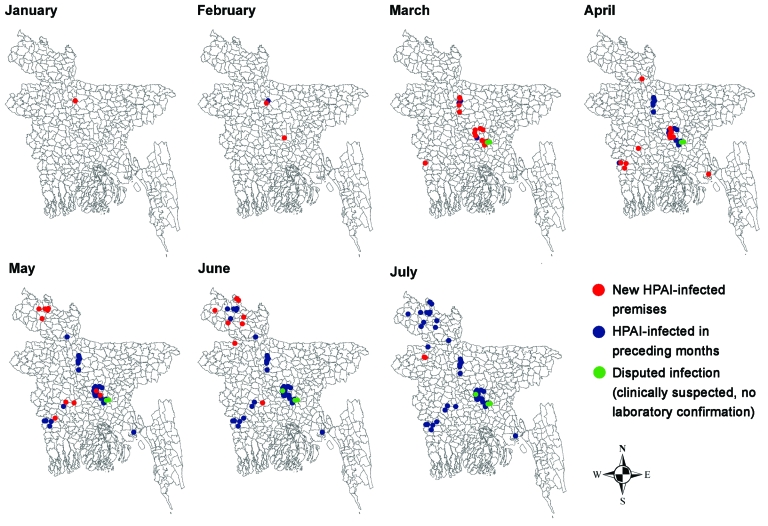
Monthly spread of highly pathogenic avian influenza (HPAI) outbreaks in chickens, Bangladesh, January–July, 2007.

We found that the H cleavage site of the selected influenza subtype H5N1 isolates (determined from the Veterinary Laboratory Agency) contained polybasic amino acids, which are characteristic of HPAI A viruses (Table 2). According to categories established by the Food and Agriculture Organization (*5*), 5 breeder and 2 layer farms had production system 2, 28 layer farms and 1 broiler farm had system 3, and 20 backyard farms had system 4. The 7-day rolling means of the numbers of avian influenza–affected farms are shown in Figure 1; the temporal and spatial spreads, in Figure 2. The index farm was recorded on January 15, 2007, at a local live bird market in Sarishabari *upazila* in Jamalpur district. We hypothesized that the infection probably came from chickens in nearby backyard farms because high numbers of deaths in this population went uninvestigated.

**Table 2:** Samples sent to the Veterinary Laboratory Agency, UK, during outbreaks of highly pathogenic avian influenza, Bangladesh, 2007*

Month†	No. samples sent	Influenza virus subtype, no. positive		Hemagglutinin cleavage site motif
		H5N1	H9N2	
Jan	0	NA	NA	NA
Feb	0	NA	NA	NA
Mar	0	NA	NA	NA
Apr	13	12	1	PQGERRRKKRGLF
May	10	9	1	PQGERRRKKRGLF
Jun	8	6	1	PQGERRRKKRGLF
Jul	6	5	NA	NA
Total	37	32	3	NA

*NA, not applicable. †Month when the first clinical outbreak was reported.

The outbreaks peaked on March 26, 2007, when 11 affected farms in 3 districts—Dhaka, Gazipur, and Narayangogj—formed a cluster, indicating a common source. The source may have been larger live bird markets, which probably infected chickens of the 15 districts. The first avian influenza–affected backyard farm was reported on March 22, 2007, the date when avian influenza was confirmed in Bangladesh. Of the 20 backyard farms, 14 were in 7 northern districts.

The overall attack rates for the *upazilas* of the infected districts were 6/1,000 commercial farms and 1/100,000 backyard farms (Table 1). Uninvestigated deaths of backyard chickens could result in underestimation of the attack rate.

Among the 9 probable sources of infection, egg trays and contaminated vehicles from larger live bird markets and local live bird markets accounted for 47% of probable virus sources, eggs for 48%, and apparently healthy chickens for 5%. One avian influenza–affected farm disposed of ≈1,000 dead chickens in an open field before diagnosis was confirmed. For the backyard chickens, sources of spread were selling chickens (5%), giving chickens to relatives or neighbors (15%), moving birds through local poultry vendors, and hiding birds during culling operations (10%).

On the index farm, chickens in 1 shed were infected, but chickens in 2 other sheds <40 yards away remained clinically unaffected through the time of culling (71 days after clinical onset). Although the media reported that a corporate-run poultry farm, Biman Poultry Complex, was the first avian influenza–affected farm in Bangladesh, our investigation found it to be the third. Of the 5 breeder farms, 2 had imported chicks from the United States and 1 from the United States and France, but these chicks had arrived >21 days before clinical onset of HPAI.

On May 22, 2008, the Directorate General of Heath Services, Bangladesh, declared that a sample collected from a child in January 2008 was diagnosed by the US Centers for Disease Control and Prevention as positive for influenza virus (H5N1). Before this time, no human infection with influenza virus (H5N1) had been reported in Bangladesh. Lack of human cases may have resulted from early immunologic response (*6*,*7*), genetic variation in receptors (*8*–*10*), poor surveillance of disease in humans, or using antiviral drugs during culling of birds.

## Conclusions

Our investigation showed that the epicenter of the HPAI outbreaks in Bangladesh was the Sarishabari *upazila* of Jamalpur district and that the primary source of infection was backyard chickens. Phylogenetic analysis on 1 influenza virus (H5N1) isolate showed that it belongs to the subclade 2.2 of the Qinghai lineage (*11*), most closely related to viruses isolated from Afghanistan, Mongolia, and Russia (*11*). Therefore, the virus might have entered Bangladesh through migratory birds (*12*–*14*). The presence of influenza virus subtype H9N2 in chickens on 3 farms, however, raises the question of when this virus was introduced to Bangladesh. An earlier introduction or emergence of LPAI virus (H9N2) in backyard chickens cannot be ruled out because ≈18% of backyard chickens tested during 2000–2003 were seropositive for avian influenza virus (*15*).

This study illustrates the progression of HPAI in Bangladesh. Further study is needed to provided more evidence for the sources we have identified.
